# Higher abundance of *Campylobacter* in the oral microbiome of Japanese patients with moyamoya disease

**DOI:** 10.1038/s41598-023-45755-3

**Published:** 2023-10-29

**Authors:** Kai Takayanagi, Fumiaki Kanamori, Kazuki Ishii, Kinya Yokoyama, Yoshio Araki, Masaki Sumitomo, Sachi Maeda, Shunsaku Goto, Shinji Ota, Yuichi Nagata, Masahiro Nishihori, Satoshi Maesawa, Takashi Izumi, Syuntaro Takasu, Ryuta Saito

**Affiliations:** 1 Department of Neurosurgery, Nagoya University Graduate School of Medicine, 65 Tsurumai, Showa-ku, Nagoya, Aichi 466-8550 Japan; 2 Department of Neurosurgery, Japanese Red Cross Aichi Medical Center Nagoya Daini Hospital, Nagoya, Japan; 3 Department of Neurosurgery, Toyota Kosei Hospital, Toyota, Japan; 4 Department of Neurosurgery, Handa City Hospital, Handa, Japan; 5 Department of Neurosurgery, Japan Community Health Care Organization Chukyo Hospital, Nagoya, Japan

**Keywords:** Stroke, Microbiome

## Abstract

Genetic factors alone cannot explain the pathophysiology of moyamoya disease (MMD), and environmental factors such as an immune response are thought to be involved. Oral and gut microbiomes have attracted attention as environmental factors in the pathophysiology of some vascular and autoimmune diseases. However, the relationship between MMD and these microbiomes is yet to be thoroughly investigated. This prospective case–control study aimed to compare the microbiomes of Japanese patients with MMD with those of healthy individuals to identify the specific bacteria involved in MMD. Saliva and fecal samples were collected from 16 patients with MMD who had not undergone revascularization surgery. Fifteen healthy individuals were matched for age, sex, and body mass index. The microbiomes were determined using 16S rRNA sequencing and analyzed using QIIME2. Differentially abundant microbes were identified using LEfSE and ANCOM-BC. In the oral microbiome, the two analytical methods showed that *Campylobacter* was more abundant in patients with MMD than in healthy individuals. Differences in the gut microbiome were smaller than those in the oral microbiome. In conclusion, the oral microbiome profiles of patients with MMD significantly differ from those of healthy individuals. *Campylobacter* spp. could be a substantial environmental factor in the pathophysiology of MMD.

## Introduction

Moyamoya disease (MMD) is a rare cerebrovascular disease characterized by stenosis in the terminal portions of the bilateral carotid arteries and collateral blood vessels due to compensatory mechanisms corresponding to that stenosis^1^. Inadequate collateral blood vessel development causes cerebral ischemia in pediatric patients with MMD, and fragile collateral blood vessels drive intracranial hemorrhage in adult patients with MMD^2^. The only established treatment to reduce the risk of cerebral ischemia or intracranial hemorrhage is revascularization surgery performed by neurovascular surgeons^3^.

Although *Ring Finger Protein 213 (RNF213)* was identified as a susceptibility gene for MMD in 2011^4,5^, *RNF213* alone cannot explain its pathophysiology. The *RNF213* p.R4810K variant is estimated to be present in 2.5% of the general Japanese population; however, less than 1% of these individuals develop MMD^6^. Therefore, environmental factors are thought to be involved in the pathogenesis of MMD, and an immune response is the most promising candidate. Pathology findings indicate that proliferating smooth muscle cells and inflammatory cells (macrophages and T cells) are colocalized in the thickened intima of occlusive major intracranial arteries^7^. In addition, transcriptome-wide analysis of blood and intracranial artery samples from patients with MMD shows that the pathophysiology of MMD is related to the immune response^8,9^.

The oral and gut microbiomes have attracted attention because of their association with vascular and autoimmune diseases^10–15^. The gut microbiome of patients with atherosclerotic cardiovascular disease deviates from that of healthy controls^10^. In cerebrovascular diseases, CNM-positive *Streptococcus mutans* in the oral microbiome is associated with an increased incidence of cerebral microbleeds^11^, and *Campylobacter ureolyticus* in the gut microbiome may be related to the rupture of cerebral aneurysms^12^. Regarding autoimmune diseases, the status of inflammatory bowel disease is strongly correlated with the intestinal immune system, which is activated by intestinal colonization of bacteria from oral origin^13,14^. In rheumatoid arthritis, dysbiosis of the oral and gut microbiomes promotes the production of autoantibodies that migrate to the joints and contribute to disease onset^15^.

MMD is a cerebrovascular disease in whose pathophysiology the immune response plays an essential role. However, the relationship between MMD and these microbiomes remains unelucidated^16^. We suspect that a particular bacterium in the oral or gut microbiome drives the immune response that causes MMD. This study aimed to investigate whether there is a difference in the oral and gut microbiome profiles between patients with MMD and healthy individuals, and to identify bacterial species specific to patients with MMD by 16S rRNA sequencing.

## Results

### Characteristics of the study cohort

The characteristics of the study cohort for each sample are summarized in Table 1. For saliva samples, the median patient age was 32 years (IQR 25–39; range 7–58) for healthy controls and 32 years (IQR 10–45; range 5–55) for patients with MMD. For fecal samples, the median patient age was 32 years (IQR 25–39; range 4–58) for healthy controls and 34 years (IQR 17–46; range 5–55) for patients with MMD. Demographic data, including sex, body mass index, hypertension, dyslipidemia, diabetes, smoking, and alcohol consumption, were also similar between groups with no significant differences.

**Table 1:** The characteristics of the study cohort in saliva samples and fecal samples.

Characteristics	Saliva samples		p-value	Fecal samples		p-value
	Control, n = 15	MMD, n = 16		Control, n = 15	MMD, n = 15	
Age, median (IQR, range)	32 (25–39, 7–58)	32 (10–45, 5–55)	0.97	32 (25–39, 4–58)	34 (17–46, 5–55)	0.65
Female, n (%)	7 (46.7)	7 (43.8)	0.87	6 (40.0)	7 (46.7)	0.71
BMI, median (IQR)	22.4 (19.3, 24.2)	23.4 (19.5, 26.3)	0.35	22.4 (19.3, 24.0)	23.8 (20.8, 26.5)	0.25
HT, n (%)	1 (6.7)	3 (18.8)	> 0.99	1 (6.7)	2 (13.3)	0.9
DL, n (%)	1 (6.7)	3 (18.8)	0.6	1 (6.7)	3 (20.0)	0.6
CKD, n (%)	0 (0.0)	0 (0.0)	1.0	0 (0.0)	0 (0.0)	1.0
Diabetes, n (%)	0 (0.0)	0 (0.0)	1.0	0 (0.0)	0 (0.0)	1.0
Smoking, n (%)	0 (0.0)	2 (12.5)	0.48	0 (0.0)	2 (13.3)	0.48
Drinking alcohol, n (%)	2 (13.3)	3 (18.8)	> 0.99	2 (13.3)	3 (20.0)	> 0.99

Continuous variables are shown as medians and categorical variables as n (%).

*BMI* body mass index, *CKD* chronic kidney disease, *DL* dyslipidemia, *HT* hypertension, *IQR* interquartile range.

### Differences in oral microbiome between patients with MMD and healthy people

**Figure 1:**
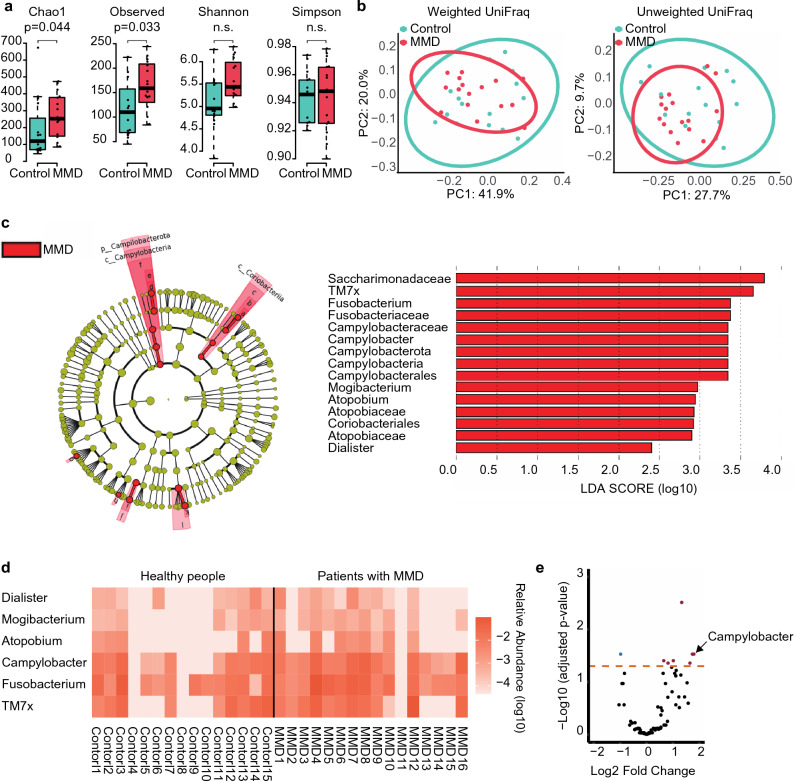
Comparison of oral microbiome between patients with MMD and healthy people. (**a**) Box and beeswarm plots showing alpha diversity. The Chao1 index and observed species were significantly different between the MMD and control groups. (**b**) Principal coordinate analysis plots of beta diversity based on weighted and unweighted UniFrac distances. There was no difference between the MMD and control groups in weighted UniFrac distances (p = 0.779) or unweighted UniFrac distances (p = 0.181). (**c**) Cladogram showing discriminative taxa identified with default parameters in LEfSe (p < 0.05, linear discriminant analysis score (LDA) > 2.0). *Campylobacter* was enriched in patients with MMD at both the phylum and genus levels. The red bar shows the LDA score for one phylum, two classes, two orders, four families, and six genera that were more abundant in patients with MMD. (**d**) Heatmap of the discriminative genera identified using LEfSe. These taxa were more abundant in patients with MMD. (**e**) Volcano plot of the values calculated using ANCOM-BC. *Campylobacter* had a significantly lower adjusted p-value (< 0.05) with a higher fold change.

For alpha diversity, the Chao1 index and observed species demonstrated significant differences between groups (Fig. 1a). No differences in beta diversity were observed between the groups (Fig. 1b).

In the linear discriminant analysis (LDA) effect size (LEfSE), one phylum (*Campylobacterota*), two classes (*Campylobacteria*, *Coriobacteriia*), two orders (*Campylobacterales*, *Coriobacteriales*), four families (*Saccharimonadaceae*, *Fusobacteriaceae*, *Campylobacteraceae*, *Atopobiaceae*), and six genera (*TM7x*, *Fusobacterium*, *Campylobacter*, *Mogibacterium*, *Atopobium*, *Dialister*) showed differentially abundant microbes between groups (Fig. 1c,d). Notably, *Campylobacter* was more abundant at the phylum and genus levels in patients with MMD. In the analysis of the compositions of microbiomes with bias correction (ANCOM-BC), ten genera containing *Campylobacter* were differentially abundant between the groups (Fig. 1e, Table 2).

**Table 2:** Differentially abundant genera in the oral microbiome between patients with MMD and healthy individuals.

Genus	log2FC (MMD/health)	p-value	Adjusted p-value
*Mogibacterium*	1.33	0.000031	0.0031
*Campylobacter*	1.8	0.0015	0.029
*Candidatus saccharimonas*	1.74	0.0015	0.029
*Saccharibacteria*	2.27	0.0015	0.029
*Streptococcus*	0.65	0.0028	0.039
*Dialister*	1.02	0.0026	0.039
*Actinomyces*	2.15	0.0045	0.044
*Veillonella*	0.81	0.0044	0.044
*Fusobacterium*	1.65	0.0039	0.044
*F0332*	− 0.98	0.0007	0.029

### Comparison of gut microbiome between patients with MMD and healthy people

**Figure 2:**
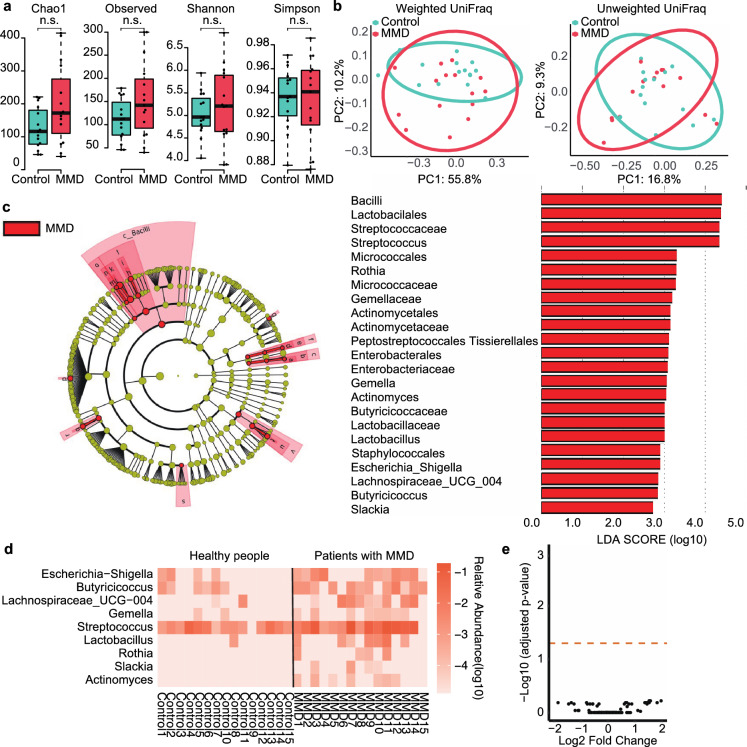
Comparison of gut microbiome between patients with MMD and healthy people. (**a**) Box and beeswarm plots showing alpha diversity. No significant differences were observed between the groups. (**b**) Principal coordinate analysis plots for beta diversity. There was no difference between the MMD and control groups in the weighted UniFrac distances (p = 0.456) and unweighted UniFrac distances (p = 0.399). (**c**) Cladogram showing the discriminative taxa identified using the default parameters in LEfSe. *Streptococcus* and *Lactobacillales* were enriched in patients with MMD. The red bar shows the LDA score for one class, five orders, eight families, and nine genera that were more abundant in patients with MMD. (**d**) Heatmap of the discriminative genera identified using LEfSe. These taxa were more abundant in patients with MMD. (**e**) Volcano plot of the values calculated using ANCOM-BC. None of the genera showed a low adjusted p-value (p < 0.05).

Differences between the groups were not observed in alpha or beta diversity (Fig. 2a,b).

In LEfSE, one class (*Bacilli*), five orders (*Lactobacilales*, *Micrococcales*, *Actinomycetales*, *Enterobacterales*, *Staphylococcales*), eight families (*Streptococcaceae*, *Micrococcaceae*, *Gemellaceae*, *Actinomycetaceae*, *Peptostreptococcales Tissierellales*, *Enterobacteriaceae*, *Butyricicoccaceae*, *Lactobacillaceae*), and nine genera (*Streptococcus*, *Rothia*, *Gemella*, *Actinomyces*, *Lactobacillus*, *Escherichia_Shigella*, *Lachnospiraceae_UCG_004*, *Butyricicoccus*, *Slackia*) showed differentially abundant microbes between groups (Fig. 2c,d). However, no differentially abundant microbes were identified in ANCOM-BC (Fig. 2e).

## Discussion

We investigated whether patients with MMD have specific oral and gut microbiomes compared to healthy individuals based on the assumption that these microbiomes are involved in the immune response in MMD. The 16S rRNA data showed that patients with MMD had different oral microbiomes, especially *Campylobacter*, than healthy controls. In addition, differences in the gut microbiome between patients with MMD and healthy individuals were smaller than those in the oral microbiome.

For the first time, this study revealed differences in alpha diversity (Chao1 index, observed species) and differentially abundant microbes in the oral microbiome. As the Chao1 index and observed species quantified the richness of species, patients with MMD had a greater diversity of oral bacteria. Furthermore, both analytical methods for detecting differentially abundant microbes consistently demonstrated that *Campylobacter* was significantly more abundant in patients with MMD. Oral bacteria can transiently access the bloodstream during daily oral hygiene practices^17^, and certain oral bacteria (*Porphyromonas gingivalis*) have been reported to infiltrate the brain^18^. In addition, although it is not a vascular cell line, *Campylobacter* modulates the immune response by upregulating TLR4 and MD-2 in HT-29 cells (a cell line with epithelial morphology)^19^. Therefore, the relationship between these mechanisms of *Campylobacter* (transient blood-borne infection and infiltration into vascular tissue) and the immune system in MMD should be investigated. Furthermore, *Campylobacter* is an intracellular bacterium containing lipopolysaccharides, and it was recently reported that RNF213 is involved in the ubiquitination of intracellular bacterial lipopolysaccharides^20^. In this regard, *Campylobacter* spp. can be considered a promising environmental factor for MMD. We suspect that the *Campylobacter* spp. may activate an immune response of the intracranial artery through transient blood-borne infection, contributing to the pathophysiology of MMD.

Regarding the gut microbiome, our results showed that differences between patients with MMD and healthy individuals were smaller than those in the oral microbiome. To date, only one study has investigated the gut microbiome of patients with MMD^16^. Our LEfSe results were similar to those of a previous report showing that *Streptococcus* and *Lactobacillales* were enriched in patients with MMD, suggesting the robustness of our study. Although a previous study showed that *Ruminococcus* is enriched in patients with MMD, our analysis did not identify this differentially abundant microbe. This difference may be because we enrolled only preoperative patients with MMD, matched control participants to patients with MMD in terms of demographic data, and used different sample collection methods.

The present study has some limitations. First, the study was conducted only in Japan. Therefore, it is essential to note regional and racial differences when comparing the current results to those of other studies. Second, the *RNF213* variant was not investigated in this study. Although *RNF213* is an important genetic factor for MMD, we considered unnecessary blood sampling to be more invasive than the study merited because our study cohort enrolled young children. Third, the sample size was relatively small. Validation of our study with a larger cohort is necessary for generalization.

## Conclusions

The oral microbiome profiles of MMD patients and healthy individuals differed significantly. *Campylobacter* spp. may be a key environmental factor in the development of MMD.

## Materials and methods

**Figure 3:**
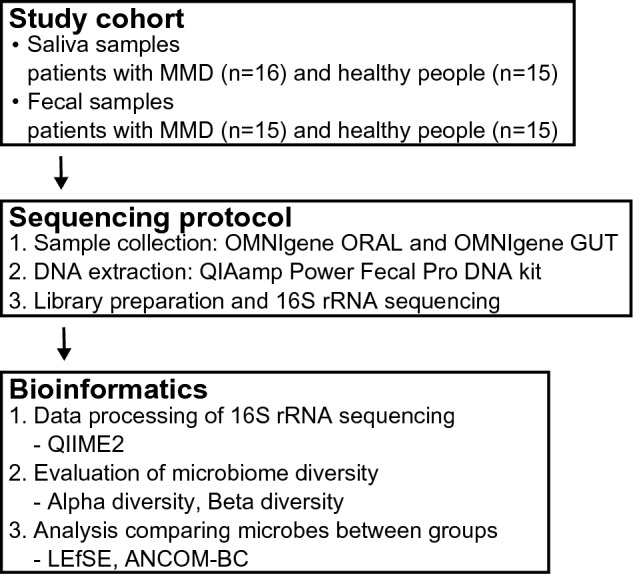
Flow diagram of the study.

A flow diagram of the study is shown in Fig. 3.

### Ethical approval and consent to participant

This study was approved by the Institutional Review Board of the Nagoya University Graduate School of Medicine (approval number 2022-0084; approval date, June 3, 2022). Written informed consent was obtained from all the participants or their legal guardians. All methods of this study were performed in accordance with relevant guidelines and regulations.

### Study cohort

We prospectively enrolled 16 patients with MMD who did not undergo revascularization surgery between June 2022 and March 2023 at the Nagoya University Hospital, Japan Community Health Care Organization Chukyo Hospital, or Toyota Kousei Hospital. The diagnosis of MMD was based on guidelines proposed by a research committee approved by the Ministry of Health, Labor, and Welfare of the Japanese government^21^. In brief, the diagnostic criteria were as follows: (1) stenosis or occlusion of the terminal portion of the intracranial internal carotid artery, (2) moyamoya vessels at the brain base, and (3) exclusion of diseases with similar angiographic characteristics (e.g., autoimmune disease, meningitis, brain tumors, Down syndrome, neurofibromatosis type 1, or cerebrovascular lesions after head irradiation). These patients did not use antibiotics within 1 month before sample collection because antibiotics affected the microbiome^22^. Of all the samples obtained from the 16 patients with MMD, one fecal sample was excluded due to inadequate collection. Finally, this study included 16 saliva and 15 fecal samples from patients with MMD.

For the healthy control group, we recruited healthy individuals who were willing to participate in our study at Nagoya University between June 2022 and March 2023. The healthy individuals had never undergone head surgery and had no history of transient ischemic attacks, ischemic stroke, or intracranial hemorrhage. Similar to the patient group, healthy individuals did not use antibiotics within one month before sample collection. Among the 17 samples collected, we selected 15 that were matched for age, sex, and BMI with the patient group.

### Sample collection methods and microbial DNA extraction experiments

OMNIgene ORAL and OMNIgene GUT (DNA Genotek, Ottawa, Canada) were used to collect the saliva and fecal samples. OMNIgene kits demonstrated good microbiome stability at room temperature within 4 days of sample collection^23,24^. Participants took these samples themselves, and we collected them within 4 days. Immediately after collection, microbial DNA was extracted from each sample using the QIAamp Power Fecal Pro DNA Kit (QIAGEN, Hilden, Germany), following the manufacturer’s instructions.

### Library preparation and 16S rRNA sequencing

Library preparation was performed according to the 16S Metagenomic Sequencing Library Preparation protocol (Illumina, San Diego, CA, USA). The V3–V4 region of the 16S rRNA microbial gene was amplified using the 16S Amplicon PCR forward primer (TCGTCGGCAGCGTCAGATGTGTATAAGAGACAGCCTACGGGNGGCWGCAG) and the 16S Amplicon PCR reverse primer (GTCTCGTGGGCTCGGAGATGTGTATAAGAGACAGGACTACHVGGGTATCTAATCC) using the KAPA HiFi HotStart ReadyMix (Kapa Biosystems, Wilmington, MA)^25^. Illumine sequencing adapters and dual-index barcodes was added to the 16S Amplicon with the Nextera XT Index kit (Illumina). Paired-end 300 base pairs sequencing was performed on the Illumina MiSeq platform using the MiSeq v3 reagent kit (Illumina). Library preparation and sequencing were performed by Macrogen Japan Corp.

### Microbiome bioinformatics

#### Data processing of 16S rRNA sequencing

16S rRNA sequencing results were analyzed using Quantitative Insights into Microbial Ecology 2 (QIIME2) v2023.2^26^. Raw sequence data were imported and demultiplexed. The generated reads were denoised into amplicon sequence variants (ASVs) using the Divisive Amplicon Denoising Algorithm 2^27^. Forward and reverse reads were trimmed, and the median quality score dropped below 20 in the quality score plot^28^. Taxonomy was assigned to the ASVs using the Scikit-learn naïve Bayes machine learning classifier against the Silva 138 99% 16S rRNA database^26,29^.

#### Evaluation of microbiome diversity

Alpha diversity was assessed by calculating the Chao1, observed species, Shannon, and Simpson indices. For statistical comparisons between patients with MMD and healthy individuals, we used the Mann–Whitney U test. Beta diversity was assessed by calculating the weighted and unweighted UniFrac distances. We performed a principal coordinate analysis (PCoA) and permutational multivariate analysis of variance (PERMANOVA). For these analyses, statistical significance was set at p < 0.05.

#### Analysis comparing microbes between MMD and control groups: LEfSE and ANCOM-BC

To identify differentially abundant microbes between groups, we performed two analytical methods up to the genus level: the LEfSE method and the ANCOM-BC^30,31^. The matching results between analytical methods increases the credibility of biological interpretation^32^. LEfSe compares the percentages of microbial composition using a non-parametric statistical test and calculates the LDA score to estimate the effect size of each differentially abundant microbe. ANCOM-BC estimated the unknown sampling fractions and corrected the bias induced by differences among samples before the statistical test. This method compared the read counts of the microbial composition using a parametric statistical test. p values were adjusted using the Benjamini and Hochberg method (adjusted p-value), and genera with an adjusted p-value < 0.05 were considered significant differentially abundant microbes^33^.

### Statistical analysis

To compare the participants’ background characteristics, numerical data were compared using the Mann–Whitney U-test, and categorical data were compared using Pearson’s Chi-squared or Fisher’s exact test. Statistical significance was set at p < 0.05. Statistical analyses were performed using the R version 4.2.2 (https://www.r-project.org/).

## Data Availability

FASTQ files of our dataset are available at the Sequence Read Archive database under the Accession Number PRJNA1011244.
